# Tryptophan metabolites in depression: Modulation by gut microbiota

**DOI:** 10.3389/fnbeh.2022.987697

**Published:** 2022-09-12

**Authors:** Iva Lukić, Sanja Ivković, Miloš Mitić, Miroslav Adžić

**Affiliations:** Laboratory of Molecular Biology and Endocrinology, Vinca Institute of Nuclear Sciences, University of Belgrade, Belgrade, Serbia

**Keywords:** depression, gut microbiota, tryptophan, serotonin, kynurenine, indole, probiotics

## Abstract

Clinical depression is a multifactorial disorder and one of the leading causes of disability worldwide. The alterations in tryptophan metabolism such as changes in the levels of serotonin, kynurenine, and kynurenine acid have been implicated in the etiology of depression for more than 50 years. In recent years, accumulated evidence has revealed that gut microbial communities, besides being essential players in various aspects of host physiology and brain functioning are also implicated in the etiology of depression, particularly through modulation of tryptophan metabolism. Therefore, the aim of this review is to summarize the evidence of the role of gut bacteria in disturbed tryptophan metabolism in depression. We summed up the effects of microbiota on serotonin, kynurenine, and indole pathway of tryptophan conversion relevant for understanding the pathogenesis of depressive behavior. Moreover, we reviewed data regarding the therapeutic effects of probiotics, particularly through the regulation of tryptophan metabolites. Taken together, these findings can open new possibilities for further improvement of treatments for depression based on the microbiota-mediated modulation of the tryptophan pathway.

## Introduction

Clinical depression is one of the leading causes of disability in modern society, affecting 5% of adults according to the latest WHO data (GHDx, 2022). The symptoms of depression include feelings of sadness and hopelessness, anhedonia, fatigue, changes in appetite, and cognitive and sleep problems (American Psychiatric Association, 2013). Depression is often comorbid with symptoms of anxiety (Fava et al., 2000; Belzer and Schneier, 2004). It is a multifactorial disorder caused by a complex interaction of genetic and environmental factors, with multiple biological systems shown to be disturbed, including neurotransmitter balance, immune response, stress response, and neuroplasticity (Nestler et al., 2002; Krishnan and Nestler, 2008).

Accumulated evidence from recent years has revealed one more important player in the etiology of depression. That is the community of an immense number of microorganisms lying at the junction of the environment and the host organism, with the greatest abundance in the gut, consisting of more than 10^13^ organisms and 10^6^ genes (Sender et al., 2016; Tierney et al., 2019). There is an intensive and bidirectional communication between gut microbiota and the brain, referred to as the gut microbiota-brain axis, affecting our behavior and mood. This complex interaction is achieved in multiple ways involving neural, immune, and endocrine systems, as well as by the direct impact of microbial metabolites on the host cells (Cryan and Dinan, 2012; Morais et al., 2020).

Numerous preclinical and clinical data have confirmed that gut microbiota dysbiosis is related to depressive behavior (De Palma et al., 2015; Kelly et al., 2016; Zheng et al., 2016; Li et al., 2019; Lukić et al., 2019a). Growing data from animal and human studies show that administration of different probiotics (usually *Lactobacillus* and *Bifidobacterium* species with beneficial effects) could alleviate symptoms of depression (Benton et al., 2007; Bravo et al., 2011; Marin et al., 2017; Pinto-Sanchez et al., 2017; Tian et al., 2019). Gut bacteria composition may influence depression by modulating the functioning of different systems that are disturbed in this disorder, including tryptophan metabolism, immune system, and neuroplasticity (Cryan and Dinan, 2012; Morais et al., 2020).

The focus of this review is the dysregulation of tryptophan metabolism, which has been implicated in depression for more than 50 years. Tryptophan is best known as a precursor of the neurotransmitter serotonin, one of the first biochemical compounds shown to be disturbed by depression (Coppen, 1967). However, as subsequent studies revealed, other tryptophan catabolites, such as kynurenine and its metabolites, also have neuroactive properties, affecting the development of depression (Oxenkrug, 2013; Dantzer, 2016). Gut microbiota could shape tryptophan metabolic pathways in multiple ways, *via* direct or indirect mechanisms, modulating host physiology and behavior, including functioning of immune system, gastrointestinal tract, metabolic processes, as well as neurodevelopment, anxiety and depressive behavior (Agus et al., 2018; Gheorghe et al., 2019; Bosi et al., 2020). Therefore, tryptophan metabolism emerged as an important hub in bidirectional gut microbiota-brain communication that can contribute not only to the development of depression but also to the treatment outcomes in depressed patients. Accordingly, the aim of this review was to summarize accumulated data of important roles that gut microbes have in the regulation of tryptophan metabolism, promoting the development of depressive behavior. Moreover, we reviewed data regarding the therapeutic effects of probiotics which exert their antidepressant actions through the modulation of tryptophan metabolism, thus opening new opportunities for improvement of treatments for depression.

## Tryptophan metabolism

**FIGURE 1:**
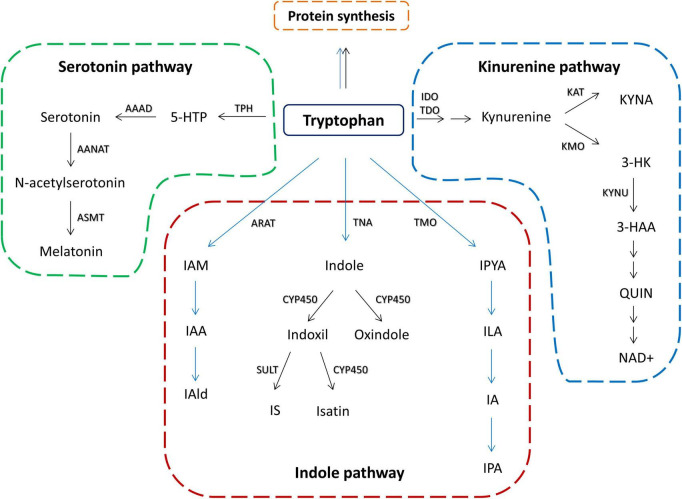
Schematic overview of tryptophan metabolic pathways in the host and its commensal bacteria. Black arrows represent host metabolism. Blue arrows represent microbial metabolism. Serotonin pathway – TPH, tryptophan hydroxylase; 5-HTP, 5-hydroxytryptophan; AAAD, aromatic amino acid decarboxylase; AANAT, aralkylamine *N*-acetyltransferase; ASMT, acetylserotonin *O*-methyltransferase; kynurenine pathway – IDO, indolamine 2,3-dioxygenase; TDO, tryptophan 2,3-dioxygenase; KAT, kynurenine aminotransferase; KYNA, kynurenine acid; KMO, kynurenine 3-monooxygenase; KYNU, kynureninase; 3-HK, 3-Hyroxykynurenine; 3-HAA, 3-hydroxyanthranilic acid; QUIN, quinolinic acid; NAD+, nicotinamide adenine dinucleotide; indole pathway: ARAT, aromatic amino acid aminotransferase; IAM, indole-3-acetamide; IAA, indole acetic acid; IAld, indole-3-aldehyde; TNA, tryptophanase; CYP450, cytochrom P450 enzymes; SULT, sulfotransferase; IS, indoxil-3-sulfate; TMO, tryptophan 2-monooxygenase; IPYA, indole-3-pyruvate; ILA, indole-3-lactic acid; IA, indole acrylic acid; IPA, indole-3-propionic acid.

Tryptophan is one of the essential amino acids and it is mainly obtained from food. Although some bacterial species are shown to produce tryptophan, such as *E. coli* (Zhao et al., 2012), there are no data that bacterial tryptophan synthesis can significantly contribute to the host tryptophan resources. In the mammalian organism, besides its role in protein biosynthesis, tryptophan can be converted along two main pathways: (1) serotonin pathway, leading to the production of serotonin and melatonin, and (2) kynurenine pathway leading to the production of kynurenine and its downstream metabolites (Yao et al., 2011; Figure 1). Additionally, there is an indole pathway, active in certain commensal gut microorganisms (Figure 1), in which tryptophan is converted to indole and its derivatives that can exert biological effects at the host organism (Lee and Lee, 2010; Zelante et al., 2013).

Upon digestion of the food, tryptophan is absorbed in the intestine to serve as a metabolic source to the host cells, including the nervous system cells, or stays available to the commensal bacteria for their metabolic activities. In the blood, majority of tryptophan, 85–90%, is bound to albumin, while only free tryptophan is available for metabolism and transport to the brain (Ruddick et al., 2006). Its transport across the blood-brain barrier (BBB) is mediated by the L-amino acid transporter (LAT) system, and in the brain, it becomes available for neurons and glial cells for their metabolic pathways (Ruddick et al., 2006). Additionally, some of the tryptophan metabolites (see later) produced at the periphery can also be transported to the brain *via* different mechanisms. Besides the direct effects of tryptophan metabolites on the central nervous system (CNS), they can affect brain functioning indirectly *via* immune or neural routes (see later).

### Serotonin pathway

Tryptophan is converted down the serotonin pathway by the activity of tryptophan hydroxylase (TPH, there are two variants, TPH1 and TPH2), which is a rate-limiting enzyme of the pathway (Boadle-Biber, 1993; Walther et al., 2003). The resulting product, 5-hydroxytryptophan (5-HTP), is further converted to serotonin (or 5-hydroxytryptamine, 5-HT) by the activity of tryptophan decarboxylase. There are two distinct pools of serotonin in the body, one is in the brain, and the other is at the periphery. The main source of serotonin in the brain are neurons of the raphe nucleus in the midbrain, expressing TPH2, from which terminals serotonin is released to virtually all parts of the brain, modulating different aspects of behavior, including mood, cognition, appetite, and sleep (Jacobs and Azmitia, 1992). However, more than 90% of serotonin is synthesized in the gut by enterochromaffin cells, expressing TPH1, which is a source of peripheral serotonin (Mawe and Hoffman, 2013).

In the pineal gland, serotonin is further converted to melatonin through the actions of arylalkylamine-*N*-acetyltransferase (AANAT) and acetylserotonin *O*-methyltransferase (ASMT) (Klein, 2007). Its synthesis is under the control of the suprachiasmatic nucleus (SCN) of the hypothalamus, which activity mediates dark-light information and is regulated by clock genes (Maronde and Stehle, 2007). Melatonin is subsequently released into the bloodstream and orchestrates the biological rhythms of the body. Apart from the pineal gland, melatonin can be synthesized by all cells containing mitochondria, including enterochromaffin cells of the gastrointestinal tract (Bertrand et al., 2014) and immune cells (Calvo et al., 2013; Carrillo-Vico et al., 2013), and can act locally exerting anti-inflammatory and antioxidant effects.

### Kynurenine pathway

The kynurenine pathway is a dominant way of catabolizing tryptophan in the body, utilizing nearly 90% of this amino acid (Yao et al., 2011) and leading to the synthesis of coenzyme nicotinamide adenine dinucleotide (NAD+), essential for many biological processes. However, several neuroactive kynurenine metabolites are formed along this pathway as well. The kynurenine pathway is initiated by the transformation of tryptophan to *N*-formylkynurenine by indoleamine 2,3-dioxygenase (IDO1 and IDO2) (Thomas and Stocker, 1999; Ball et al., 2009), or tryptophan 2,3-dioxygenase (TDO) (Ren and Correia, 2000), which is further converted to kynurenine. IDO1 is a major rate-limiting enzyme of kynurenine synthesis outside the liver, including the gut and the brain (Guillemin et al., 2003, 2005), while TDO is mostly expressed in the hepatic tissue. Following the production of kynurenine, kynurenine metabolism separates along two major branches. Along the first branch, kynurenine undergoes transamination catalyzed with kynurenine aminotransferases (KATs) to form kynurenic acid (KYNA) (Schwarcz et al., 2012). Along the second branch, initiated by the enzyme kynurenine-3-monooxygenase (KMO), kynurenine is converted to 3-hydroxykynurenine (3-HK) and subsequently to quinolinic acid (QUIN) (Schwarcz et al., 2012). From the periphery, kynurenine and 3-HK readily cross BBB using LATs, and it is estimated that about 60% of CNS kynurenine metabolism is initiated by kynurenine from the systemic circulation (Gál and Sherman, 1980). On the other hand, brain levels of KYNA and QUIN depend primarily on their production in the CNS. Astrocytes are the major producers of KYNA, predominantly expressing KAT II, while microglia cells are major producers of QUIN (Heyes et al., 1996; Guillemin et al., 2000). KYNA and QUIN are major kynurenine catabolites with neuroactive properties. In the brain, they regulate the activity of the *N*-methyl-D-aspartate (NMDA) receptor, a type of glutamate receptors, which is crucial for the regulation of synaptic plasticity, but whose overactivation leads to neurotoxicity. KYNA acts as an antagonist of NMDA receptors and has a neuroprotective role, while QUIN acts as an agonist of NMDA receptors and exerts neurotoxic effects (Perkins and Stone, 1982; Schwarcz et al., 1983). Pro-inflammatory cytokines during immune system activation, especially interferon gamma (INFγ), are major inducers of IDO and enzymes of QUIN branch of kynurenine pathway, which switch the balance of kynurenine metabolism toward the excitotoxic QUIN (Campbell et al., 2014; Mithaiwala et al., 2021).

### Indole pathway

Aside from tryptophan utilization by the host cells and its conversion down the serotonin and kynurenine pathways, the resident microbiota can metabolize tryptophan to another biomolecule called indole that has important roles in processes essential for bacterial communication and survival, but also influences human physiology. It is estimated that about 5% of ingested tryptophan is utilized by gut microbes in the large intestine for their metabolism (Hyland et al., 2022). The hydrolysis of tryptophan in the indole pathway is performed by the enzyme tryptophanase, which is expressed in many bacteria, including *Escherichia coli*, *Clostridium* spp., and *Bacteroides* spp. (Smith and Macfarlane, 1996; Lee and Lee, 2010). Tryptophanase is encoded by the bacterial *tnaA* genes, and up to date no eukaryotic cells are shown to produce indole (Lee and Lee, 2010). After being expelled by bacterial cells, indole can be absorbed by the host cells and converted by hepatic xenobiotic metabolizing enzymes, cytochromes P450, and sulfotransferases, into several compounds, including indoxyl-3-sulfate (IS), the most dominant host indole metabolite, as well as oxindole and isatin (King et al., 1966; Gillam et al., 2000; Banoglu and King, 2002; Andriamihaja et al., 2022). Tryptophan can also be degraded into various indolic compounds by intestinal bacteria depending on the catalytic enzymes they harbor, such as indole-3-propionic acid (IPA), indole-3-acetaldehyde (IAld), indole-3-acetic acid (IAA), indole-3-lactic acid (ILA) and many others (Roager and Licht, 2018). The production of indole and its derivatives varies between different individuals as a result of diverse gut microbial communities they harbor (Jaglin et al., 2018). Therefore, the existence of differences in individuals’ gut microbiomes, and its disturbances, could result in excessive or diminished production of indole and its derivatives, which can further significantly influence human physiology, including regulating the function of the gastrointestinal tract and immune system, as well as brain and behavior.

## Serotonin, depression, and microbiota

### Serotonin in depression

The serotonin hypothesis was one of the first biological theories of depression, introduced by Coppen more than 50 years ago, which was based on the mechanism of action of some of the first tricyclic antidepressants (Coppen, 1967; Albert et al., 2012). The major evidence showing the contribution of the serotonergic system to the pathology of depression comes from tryptophan depletion studies involving the acute dietary restriction of tryptophan, leading to the reduction of serotonin synthesis (Jans et al., 2007). Tryptophan depletion produces acute lowering of the mood in depressed patients as well as in their first-degree relatives, but not in the healthy controls, implying that the heritable vulnerability of the serotonergic system can be a risk factor for the precipitation of depression (Booij et al., 2003; Ruhé et al., 2007). Furthermore, there are data about the association between depression and specific variants of genes involved in serotonin synthesis and signaling, including TPH2 (Zill et al., 2004), serotonin receptors (Lemonde et al., 2003), and serotonin transporter (SERT) (Hoefgen et al., 2005; Avshalom et al., 2010).

The most commonly used drugs for the treatment of depression act to increase serotonin signaling in the brain and include selective serotonin reuptake inhibitors (SSRIs) as well as serotonin norepinephrine reuptake inhibitors (SNRIs) (Frazer, 1997; Rush et al., 2004). Although these antidepressants are generally considered to be efficient, there is still a high risk of relapse, indicating the need for further improvement of medication used for treating depression (Hansen et al., 2008; Reid and Barbui, 2010). There are also studies exploring tryptophan supplementation as a treatment to alleviate depression, solely or in combination with other antidepressants, as a method to increase serotonin concentration in the brain. The outcomes of these studies were conflicting, but some clinical data support the usefulness of tryptophan supplementation, at least in a subgroup of patients (Shaw et al., 2002).

### Microbiota – serotonin crosstalk in depression

**FIGURE 2:**
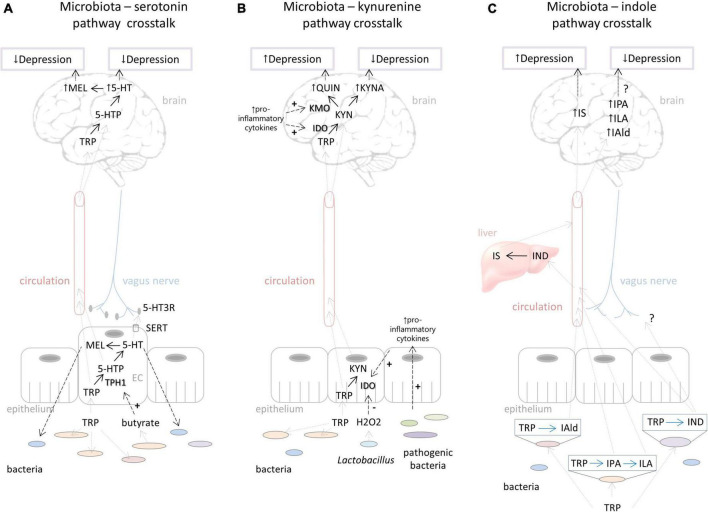
Putative ways of gut microbiota – host interactions in the context of the tryptophan metabolism influencing depressive behavior. **(A)** Effects of microbiota on serotonin pathway include regulation of tryptophan availability which directly influences serotonin and probably melatonin synthesis in the brain affecting depressed mood. Microbiota can stimulate TPH1 activity by its metabolites (e.g., butyrate). Serotonin produced in the gut could have an impact on brain functioning *via* stimulation of the vagus nerve. On the other hand, host serotonin and melatonin synthesis in the gut can affect the commensal bacterial composition, contributing individual’s susceptibility to depression. **(B)** Bacterial effects on the kynurenine pathway include down-regulation of IDO activity by H_2_O_2_ produced by *Lactobacillus*. The proliferation of pathogenic bacteria that stimulates pro-inflammatory cytokine production stimulates IDO activity and QUIN branch of the kynurenine pathway. Increased QUIN is associated with depression. On the other hand, increased KYNA levels could be related to reduction of depressive symptoms. **(C)** Depending on the specific catalytic enzymes that different bacterial species harbor, tryptophan can be degraded into various indolic compounds. They are absorbed by the host into the circulation and can be further metabolized in lever; for example, indole is metabolized to IS, which could, in high concentrations, lead to depressive behavior. Various indolic compounds (e.g., IPA, ILA, IAld) were shown to have neuroprotective effects, but their effects on depression are still unexplored. For more details, see explanations in the text. Black dashed arrows represent directions of effects (“+” - represents stimulation, “-” - represents inhibition). Gray dashed arrows represent directions of movement of metabolites. Black dotted arrows represent effects on depression. Black full arrows represent host pathways. Blue full arrows represent bacterial pathways. Question marks represent effects that are not still well explored. TRP, tryptophan; TPH, tryptophan hydroxylase; 5-HTP, 5-hydroxytryptophan; 5-HT, 5-hydroxytryptamine (serotonin); MEL, melatonin; SERT, serotonin transporter; EC, enterochromaffin cells; IDO, indolamine 2,3-dioxygenase; TDO, tryptophan 2,3-dioxygenase; KYN, kynurenine; KYNA, kynurenine acid; KMO, kynurenine 3-monooxygenase; QUIN, quinolinic acid; IAld, indole-3-aldehyde; IPA, indole-3-propionic acid; ILA, indole-3-lactic acid; IND, indole; IS, indoxil-3-sulfate.

The availability of blood tryptophan is the major limiting factor of serotonin synthesis in the brain (Figure 2A). Germ free (GF) mice, which are depleted from all commensal microorganisms in the body, have elevated tryptophan levels in the blood and brain (Wikoff et al., 2009; Clarke et al., 2013; Lukić et al., 2019a). This is expectable since gut bacteria, use tryptophan for their metabolic processes, and in the absence of bacteria more tryptophan is available to the host. Accordingly, GF mice exhibit higher serotonin levels in the brain, e.g., prefrontal cortex and hippocampus (Clarke et al., 2013; Lukić et al., 2019a). These elevated levels of brain serotonin were related to decreased depressive behavior of GF mice (Lukić et al., 2019a). However, after acute tryptophan reduction, the depressive behavior of GF mice increased and was comparable to the depressive behavior of their counterparts with normal microbiota. Furthermore, the increase in depressive behavior of GF mice was much stronger than in their counterparts, plausibly as a result of a stronger reduction of tryptophan and serotonin levels in the hippocampus and the prefrontal cortex (Lukić et al., 2019a). Therefore, the serotonergic system of GF mice is more sensitive to fluctuations of available tryptophan than in the conventional mice. We could conclude that normal gut microflora, although lowering tryptophan levels available to the host, has protecting effects toward buffering its serotonergic system to be less sensitive to variations of its precursor.

In addition, Clarke et al. (2013) showed that perturbations of the serotonin system in GF mice could not be restored by reestablishing the microbiological environment during early adolescence, implying a developmental window in which gut microbes can exert their effects on the maturation of the serotonergic system. It would be compelling to explore in future studies which specific bacteria affect serotonin system maturation making it more or less vulnerable to depression. Also, the same authors found that increased circulating levels of tryptophan, as well as higher serotonin synthesis in the hippocampus of GF mice, were observed only in males but not in females (Clarke et al., 2013). Therefore, sex hormones seem to be implicated in the microbial roles in tryptophan metabolism and the shaping of the serotonergic system in the brain (Jašarević et al., 2016; Yoon and Kim, 2021). This observation is worth further exploration since females are two times more prone to depression than males (GHDx, 2022).

The serotonin system in the gut, which is under the control of certain commensal bacteria, could also play an important role in the regulation of brain functioning and behavior (Figure 2A). Yano et al. (2015) showed that spore-forming bacteria directly influence serotonin synthesis by stimulating TPH1 expression in the enterochromaffin cells *via* their particular metabolites, including butyrate, deoxycholate, and cholate. Gut serotonin could signal to the brain *via* the neural route, i.e., by activating serotonin receptors at terminals of vagal afferents that communicate to the neurons of the brainstem (Browning, 2015). Interestingly, the antidepressant effects of SSRIs, which increase available serotonin, were abolished by vagotomy, indicating the important role of peripheral serotonin and vagus nerve stimulation in regulation of depressive behavior (McVey Neufeld et al., 2019). Therefore, we assume that microbial impact on serotonin synthesis in the gut could potentially influence depressive behavior by stimulation of the vagus nerve, for example, but this hypothesis needs further evaluation.

Apart from the role of gut microbes in regulating host serotonin metabolism, alterations in the serotonin system of the host may, vice versa, affect its commensal bacteria communities, which in turn could contribute to the host’s susceptibility to depression. Actually, serotonin acts as interkingdom signaling molecule promoting growth and virulence in certain bacteria (Oleskin et al., 1998; Knecht et al., 2016). Rodents with the absence of TPH1 enzyme (Kwon et al., 2019) or SERT (El Aidy et al., 2017) have altered gut microbial composition demonstrating that host serotonin synthesis and transport indeed affect the growth and metabolism of its resident bacteria as well. Furthermore, rats with reduced expression of SERT, when exposed to early life stress, an important etiological factor contributing to depression, exhibited grater perturbations of gut microbiota with increase in abundance of pro-inflammatory bacterial groups (El Aidy et al., 2017). These studies imply the complex bidirectional cross-talk of the host and its microbial community at the level of serotonin metabolism, which should be considered when trying to understand an individual’s susceptibility to depression.

Finally, it should be mentioned that antidepressants that target serotonergic system (SSRIs and SNRIs) affect the composition of intestinal bacteria as well, mediating at least partly their therapeutic effects. Specifically, our results demonstrated that the antidepressant effect of duloxetine (an SNRI) was abolished by supplementation with *Ruminococcus flavefacience* (Lukić et al., 2019b). *R. flavefacience* compromised antidepressant effects by diminishing serotonin levels, as well as by affecting transcription of genes involved in neural plasticity and mitochondrial metabolism of the prefrontal cortex (Lukić et al., 2019b). Therefore, SSRIs and SNRIs exert their beneficial effects on depressive behavior not only by improving host serotonin system homeostasis, but also by altering its gut microbiota composition, which in turn further contributes to the antidepressive effects of the drugs.

### Probiotic effects on serotonin in depression

Studies examining the therapeutic properties of probiotics further support the role of specific bacteria in modulating serotonin metabolism of the host, thus contributing to the improvement of depressive behavior.

Several *Lactobacillus* species exhibiting antidepressant properties in various animal models of depression were shown to increase serotonin levels. For example, Liang et al. (2015) demonstrated that *L. helveticus* can elevate hippocampal serotonin levels in parallel with reducing anhedonia, anxiety, and cognition in chronically restrained rats, the effect that was similar to citalopram, an SSRI. They suggested that the mechanisms by which *L. helveticus* achieved these effects involved an increase of anti-inflammatory mediators (interleukin 10, IL10) and a decrease of corticosterone, both known to down-regulate the kynurenine pathway, leaving more tryptophan available for serotonin synthesis (Liang et al., 2015). Further, *L. reuteri* (strain *L. reuteri 3*) ameliorated anhedonia and anxiety in mouse chronic social defeat model of depression along with increasing serotonin levels in the colon and serum (Xie et al., 2020). This probiotic bacteria increased TPH1 expression in the colon, which could contribute to the increased intestine serotonin synthesis since it is a rate-limiting enzyme of the serotonin pathway. However, the authors did not examine the serotonin levels in the brain, nor if the peripheral serotonin is indeed involved in promoting the antidepressive effect of the examined probiotic. Likewise, *L. kefiranofaciens*, isolated from kefir, improved depressive-like behavior in mice subjected to chronic unpredictable mild stress comparably to fluoxetine (an SSRI), along with elevation of serum tryptophan levels and hypothalamic serotonin (Sun et al., 2019). (Also, see later about the effects of *L. reuteri* 3 and *L. kefiranofaciens* on kynurenine pathway in parallel).

Two *Bifidobacterium* species, *B. infantis* and *B. breve* exerted antidepressive and anxiolytic effects, along with increasing levels of serotonin in the hippocampus (Tian et al., 2019). Treatment with *B. infantis* increased the cecal content of butyrate which was shown to stimulate TPH1 expression and 5-HTP secretion in the *in vitro* model of intestine enterochromaffin cells (Tian et al., 2019). The authors suggested that butyrate-stimulating 5-HTP production in the gut could supply the brain since 5-HTP readily crosses the BBB, and contribute to the increased central serotonin production, leading to the alleviation of depressive and anxiety symptoms (Tian et al., 2019). Indeed, they found a positive correlation between the levels of the gut butyrate and hippocampal 5-HTP, on the one hand, and negative relation between the gut butyrate and anxiolytic properties, on the other hand, which partly supported their hypothesis. In future research, it would also be interesting to examine whether butyrate could induce TPH2 enzyme activity in the midbrain, directly increasing serotonin production in the brain.

Finely, it is worth mentioning that some bacteria can synthesize serotonin in certain amounts *in vitro*, such as species of *Lactobacillus*, *Streptococcus, Enterococcus*, and *Escherichia* (Özoǧul et al., 2012). However, the therapeutic potential of these bacteria and the serotonin they produce is still unknown.

## Melatonin, depression, and microbiota

### Melatonin in depression

Being that melatonin is synthetized from serotonin and that serotonin deficiency was postulated to be involved in depression, it was also proposed that reduction in melatonin production could contribute to some symptoms of depression, such as disturbances in sleep and circadian rhythms (Srinivasan et al., 2006; Germain and Kupfer, 2008). Indeed, several clinical data found lower nocturnal levels of melatonin as well as a phase shift of its secretion in severely depressed patients (Brown et al., 1985; Souêtre et al., 1989; Crasson et al., 2004; Ogłodek et al., 2016; Sundberg et al., 2016). Moreover, there is evidence of antidepressant properties of melatonin supplementation (Serfaty et al., 2010; Hansen et al., 2014; Vega-Rivera et al., 2020). These beneficial effects of melatonin were shown to be achieved through reducing neuroinflammation and stimulation of neurogenesis (Ali et al., 2020; Vega-Rivera et al., 2020).

### Microbiota – melatonin crosstalk in depression

There is not much data exploring melatonin metabolism as a mediator of bacterial effects on depressive behavior. Gut bacteria could affect melatonin synthesis through regulation of tryptophan availability and serotonin synthesis, as discussed in the previous section (Figure 2A). Further, it was shown that GF mice exerted reduced amplitude in the expression of clock genes in SCN compared to their conventional counterparts, and these changes could be mediated by bacteria-derivate metabolites, such as butyrate (Leone et al., 2015). The authors showed that butyrate can directly regulate expression of circadian gens of hepatic cells *in vitro*, probably *via* its histone deacetylase (HDAC) inhibitor capacity (Leone et al., 2015). Whether such mechanism is also involved in regulation of hypothalamic clock genes needs to be conferred. Since SCN has a role in regulation of melatonin synthesis, it is feasible to assume that bacterial composition could have implications for melatonin production, but whether and how it would be reflected on depression pathogenesis remains to be explored.

It is important to mention that microbiome-derived butyrate can also up-regulate melatonin synthesis in mitochondria, and in such way optimize oxidative phosphorylation and antioxidant defense of the cells (Jin et al., 2016; Anderson and Maes, 2020). Namely, it was shown that butyrate, through inhibition of HDAC activity, leads to increased production of acetyl-CoA that is a necessary co-substrate of AANAT, converting serotonin down the melatonin pathway (Naia et al., 2017; Anderson and Maes, 2020). Since depression is linked to suboptimal mitochondrial function (Scaini et al., 2022), on one hand, and butyrate has been shown to have antidepressant properties (Caspani et al., 2019), on the other hand, it is worth examining if regulation of mitochondrial function by butyrate could contribute to alleviation of depression. Therefore, dysregulation of melatonergic pathway in mitochondria may be one more way how gut microbiota is involved in pathogenesis of depression.

Regarding the effect of melatonin on the gut microbiota, the recent study by Lv et al. (2020) showed that antidepressive properties of melatonin in dextran sulfate sodium (DSS)-induced depression in mice was accompanied with changes in gut bacteria. Namely, the authors showed that DSS increased depressive-like and anxiety behavior along with the increased inflammation in the gut and the brain, and disrupted homeostasis of gut microbiota, while melatonin treatment normalized these effects (Lv et al., 2020). Specifically, melatonin increased bacterial diversity and reduced the abundance of bacteria belonging to the class *Clostridia*, known to be associated with gut inflammation, while increasing bacteria belonging to the class *Bacilli*, including genus *Lactobacillus*, shown to have anti-inflammatory properties (Lv et al., 2020). These melatonin effects were accompanied by reduced production of pro-inflammatory cytokines and permeability of the gut and the brain (Lv et al., 2020). In several other animal models, it was also shown that melatonin could shape gut microbiota (Leone et al., 2015; Xu et al., 2017; Ren et al., 2018), but how melatonin achieves these effects remains to be elucidated. One possible mechanism includes promoting differentiation of goblet cells and production of antimicrobial peptides through TLR4 signaling (Kim et al., 2020). In addition, melatonin could promote growth of *L. johnsonii* at early stages *in vitro* and stimulate swarming motility in cultures of *Enterobacter aerogenes* (Paulose et al., 2016).

## Kynurenine metabolites, depression, and microbiota

### Kynurenine metabolites in depression

The activation of the kynurenine pathway was initially believed to enhance depressive symptoms by depleting serotonin, but later evidence emphasized that kynurenine metabolites are causally involved in the pathology of depression (Oxenkrug, 2013). The increased concentrations of peripheral kynurenine, as well as the increased kynurenine/tryptophan ratio, were found in the depressed individuals (Sublette et al., 2011; Bradley et al., 2015; Ogyu et al., 2018). Peripheral kynurenine is a relevant proxy for activation of brain kynurenine pathway since, as noted above, it readily crosses BBB and significantly contributes to kynurenine breakdown in the CNS (Haroon et al., 2020). Further, some genetic studies showed an association between enzymes of the kynurenine pathway (IDO, KMO, KAT) and liability for depression (Claes et al., 2011; Barnes et al., 2016; Boros et al., 2018; Lezheiko et al., 2018). Moreover, immunotherapies with pro-inflammatory cytokines, potent activators of IDO, could lead to the development of depressive symptoms, in about half of patients (Pollak and Yirmiya, 2002; Capuron et al., 2003; Raison et al., 2010). Finally, the role of the kynurenine pathway and IDO activation in the pathogenesis of depression was confirmed by Dancer and colleagues in a series of experiments in the rodent neuroinflammatory model of depression (O’Connor et al., 2009a,b, c). Namely, they showed that pharmacological or genetic inhibition of IDO alleviated depressive behavior of mice (O’Connor et al., 2009a,b, c) and that the activation of NMDA receptors is the main factor causing inflammation-induced depression (Walker et al., 2013). As mentioned above, KYNA and QUIN are major neuroactive kynurenine catabolites, acting on NMDA receptors, with KYNA, produced in astrocytes, being an antagonist (Birch et al., 1988) and QUIN, produced manly in microglia, an agonist (Guillemin et al., 2001). Indeed, microglial activation and increased QUIN immunoreactivity (Steiner et al., 2008, 2011; Tynan et al., 2010), on the one hand, and reduction in astrocyte number, on the other hand, were also shown in animal models of depression as well as in post-mortem brains of patients with depression (Si et al., 2004; Banasr et al., 2008; Rajkowska and Stockmeier, 2013). Therefore, the imbalance between neurotoxic and neuroprotective kynurenine metabolites may be the principal driver of depression conceivably through the alterations in glutamatergic signaling.

### Microbiota-kynurenine metabolites crosstalk in depression

The first article showing the role of microbiota in kynurenine metabolism is the study of Clarke et al. (2013), demonstrating reduced blood kynurenine/tryptophan ratio in GF mice, despite the increase of tryptophan levels. Recolonization with conventional gut microbiota during adolescence normalized circulating levels of tryptophan as well as kynurenine/tryptophan ratio, which was complemented by the normalization of anxiety behavior (Clarke et al., 2013). Therefore, the modulation of kynurenine pathway is possible through the manipulation of gut microbiota even later in life, and it is related to the regulation of at least some aspects of behavior.

Studies of fecal microbiota transplantation (FMT), as a method to investigate causative role of gut bacteria in regulating host physiology as well as behavior, demonstrated the role of gut bacteria in disturbances of kynurenine metabolism related to depressive behavior. FMT from depressed patients into the recipient rats with the depleted microbiome induced anxiety and depression-like behavior along with a significant increase in plasma kynurenine/tryptophan ratio, recapitulating the finding in the depressed patients (Kelly et al., 2016). This activation of the kynurenine pathway was parallel with a pro-inflammatory profile in the blood of depressed individuals that was partly transferred by their feces (Kelly et al., 2016). Similarly, the FMT from mice that underwent chronic mild unpredictable stress into the control recipient mice induced anxiety and depressive-like behavior accompanied by increased levels of pro-inflammatory cytokines and IDO1 expression in the hippocampus (Li et al., 2019). Further, the authors demonstrated that the levels of pro-inflammatory cytokine INFγ, a potent inducer of IDO1 (Taylor and Feng, 1991), were negatively correlated with the abundance of *Lactobacillus* species in the gut, known for their anti-inflammatory properties (Le and Yang, 2018; Mu et al., 2018). On the other hand, INFγ levels were positively correlated with abundance of *Akkermansia*, which is known for mucin degradation (Derrien et al., 2017) and can be associated with some pro-inflammatory conditions (Dodiya et al., 2020). However, this finding needs further exploration since *Akkermansia* is generally considered beneficial with anti-inflammatory properties (Cani and de Vos, 2017; Derrien et al., 2017). These studies indicated that the activation of the kynurenine pathway at the periphery and in the brain, by immune-mediated mechanism, is involved in a transfer of depressive phenotype by the gut microbiome (Figure 2B).

The role of the immune response as a mediator of gut bacteria effects at the kynurenine pathway was demonstrated in a study by El Aidy et al. (2014), in which they detected a transient increase of immune system activation after colonization of GF mice, coinciding with transient replenishment of pathobiont (*Helicobacter, Sphingomonas*, and *Mucispirillum*). This increase in pro-inflammatory cytokines was accompanied by the transient increase of intestine IDO1 expression and elevation of circulating kynurenine/tryptophan ratio (El Aidy et al., 2014). Therefore, pro-inflammatory bacteria could stimulate kynurenine synthesis in the gut indirectly through induction of immune response, contributing to the central kynurenine pool and its further catabolism.

Aside from the indirect effects of gut bacteria on kynurenine metabolism through immune system activation, an elegant study by Marin et al. (2017) demonstrated how *Lactobacillus* species could directly affect gut kynurenine production contributing to the depressive behavior of stressed mice. Namely, they showed that chronic unpredictable mild stress significantly depletes the gut of *Lactobacillus* species (Marin et al., 2017), a genus that has a capacity to produce high levels of H2O2, as a way to maintain their ecological niche. This peroxide has antimicrobial effects (Eschenbach et al., 1989; Shi and Li, 2016), but it can also down-regulate host kynurenine metabolism by inhibiting IDO1. Indeed, stressed animals exhibited decreased levels of peroxide in their feces, correlating with the reduction of *Lactobacilli* levels, reduced expression of intestine IDO1, and decreased circulating levels of kynurenine. Thus, it is possible that the reduction of *Lactobacilli* and their metabolite H2O2 leads to higher production of kynurenine in the intestine, and higher blood levels of kynurenine that readily cross BBB and further metabolized to neurotoxic products in the brain, finally contributing to the exacerbation of the depressive symptoms (Figure 2B). The authors indeed confirmed that the circulating levels of kynurenine are causally related to depressive behavior by administrating kynurenine to naïve animals and observing the increase of depressive behavior.

One more possible way with which microbiota exerts its effects on the brain kynurenine system is microRNAs (miRNAs), single-stranded non-coding RNAs that regulate eukaryotic gene expression post-transcriptionally. GF mice exhibit altered expression of miRNAs in the hippocampus, especially those that are associated with the kynurenine pathway (Moloney et al., 2017). Some of these changes were normalized by recolonization in early adolescence, although not always in the reverse manner, suggesting the complexity of the bacterial effects at miRNA expression. The exact mechanism of how microbes can influence miRNA expression in the brain is not well understood, but it can include microbial metabolites such as butyrate or lipopolysaccharide (Li et al., 2020). The possibility that behavioral effects of gut bacteria are mediated through miRNA regulating the kynurenine pathway is feasible since changes in miRNA expression in the rat brain were shown to be influenced by early life stress, a known etiological factor of depression, and reversed by antidepressants (O’Connor et al., 2013).

### Probiotic effects on kynurenine metabolites in depression

Some of the aforementioned studies of antidepressive effects of *Lactobacillus* probiotics evaluated their effects on serotonin and the kynurenine pathway in parallel. Namely, Xie et al. (2020) demonstrated that antidepressive properties of *L. reuteri* in mouse chronic social defeat model were accompanied by the reduction of IDO1 expression in the colon and PFC, while in the meantime, as previously stated, the blood and colon serotonin levels were increased. Similarly, Sun et al. (2019) showed that *L. kefiranofaciens* alleviated depression in mouse chronic unpredictable stress model with simultaneous decrease of blood kynurenine and rise of brain serotonin. Therefore, *Lactobacillus* species can exert their beneficial effects on depressive behavior by shifting tryptophan metabolism toward increasing serotonin production while inhibiting kynurenine synthesis, which could include different mechanisms. In the study of Sun et al. (2019), the changes in tryptophan metabolism caused by *L. kefiranofaciens* supplementation corresponded to a decrease of pro-inflammatory cytokines (IL6 and INFγ) as well as stress hormone corticosterone, which are known stimulants of IDO1 and TDO expression, respectively (Sun et al., 2019). Besides the potential direct effects of *L. kefiranofaciens* on the measured parameters, the observed behavioral changes could be achieved by modulating other gut bacteria species and restoring the dysbiosis caused by chronic stress (Sun et al., 2019). Namely, chronic stress decreased levels of *Bifidobacterium* and *Akkermansia*, while treatment with *L. kefiranofaciens* reversed changes in these beneficial bacteria.

Also, the abovementioned study of Marin et al. (2017) showed that administration of single species of *Lactobacillus*, i.e. *L. reuteri*, to stressed mice was able to improve their despair behavior along with improvement of metabolic homeostasis of the kynurenine pathway. Namely, *L. reuteri* production of H2O2 attenuated intestine IDO1 expression, down-regulating kynurenine production and restoring desperate behavior of stressed mice (Marin et al., 2017).

There are some data from clinical trials exploring the effects of probiotics in depressed subjects along with parameters of kynurenine pathway. Kazemi et al. (2019) showed that treatment with two probiotic bacteria *L. helveticus* and *B. longum* improved depression along with the decrease of circulating kynurenine/tryptophan ratio and an increase of tryptophan levels. Therefore, these probiotics seem to leave more tryptophan available for serotonin conversion by reducing the activity of enzymes for kynurenine synthesis. The other clinical study assessed the psychobiotic effects of *L. plantarum 299v* (LP299v) in patients with clinical depression undergoing treatment with SSRIs and showed that it decreased blood kynurenine concentrations along with the improvement of cognitive functions, although the mood was not improved (Rudzki et al., 2019). Besides already discussed ways by which *Lactobacillus* strains affect kynurenine metabolism, additional mechanism by which these bacteria could influence levels of kynurenine catabolites includes their ability to synthetize enzymatic cofactors important for certain steps of this pathway. For example, vitamin B2 is a cofactor for KMO, and *L. plantarum* strains were shown to synthesize this vitamin (Thakur et al., 2016). Indeed, the authors found that administration of *L. plantarum* correlated with the increased 3-HK/kynurenine ratio, indicating the activation of KMO. They proposed that activation of KMO could result in a detected decrease of blood kynurenine, simultaneously stimulating its conversion toward NAD+ without the accumulation of toxic kynurenines, which stayed unchanged. Unfortunately, the authors were not able to measure vitamin B levels in their study, which could be an interesting avenue for future research.

## Indole and its derivatives, depression, and microbiota

### Indole and its derivatives in depression

Regarding clinical studies, up to date, there is limited data concerning the role of indole and its metabolites in depression. Using a cohort from the large Prediction of Remission in Depression to Individual and Combined Treatments (PReDICT) study, Brydges et al. (2021) showed that plasma levels of IS were positively correlated with the severity of depression and anxiety symptoms in patients with clinical depression. As mentioned above, IS is a major product of indole degradation in the liver and is regarded as a marker of indole production by resident intestinal microbiota. Additionally, ratios of metabolites IPA/IS, and ILA/IS were negatively correlated with depression and anxiety scores. Especially, the study showed that association between IS levels and psychotic anxiety was mediated by IS effects on functional connectivity in neural networks responsible for processing aversive stimuli (Brydges et al., 2021). However, levels of measured indole metabolites were not related to the outcome of antidepressant treatments and the improvement of symptoms (Brydges et al., 2021). Further, analyses of the sample from the observational prospective NutriNet-Santé Study showed that women with recurrent depressive symptoms had significantly higher urinary IS concentrations (Philippe et al., 2021). Contrary to that, urinary metabolomics analysis of Chinese depressed patients found significantly decreased IS levels in patients with severe depression, which was one of six metabolites that could discriminate them from healthy controls with high accuracy (Chen et al., 2017). The inconsistency in the obtained results could be due to the heterogeneity of the depressive disorder itself, and other differences of analyzed samples such as gender and ethnicity, but additional clinical studies should further explore these discrepancies. One more recent study analyzed plasma levels of indole derivatives in relation to depressive symptomatology in obese patients and found reduced amounts of tryptophan and indoles, especially IAld, along with high levels of inflammatory biomarker C-reactive protein, in patients with more severe depressive symptoms (Delgado et al., 2022).

### Microbiota- indoles crosstalk in depression

Up to date, there are certain amounts of preclinical reports that confirm the role of indole and its derivatives associated with behavioral alterations in rodent models of anxiety and depression, but only beginning to uncover the potential mechanisms of these effects. The study of Jaglin et al. (2018) showed how indole physiological and behavioral effects depend on dose and duration of administration (Jaglin et al., 2018). Namely, acute injection of indole into the cecum of conventional rats, mimicking acute indole overproduction, led to neurodepressant effects and reduced motor activity accompanied by high levels of brain isatin and oxindole, oxidized indole derivatives. Indeed, other studies demonstrated that systemic injection of oxindole and isatin led to their accumulation in the brain and reduced locomotor activity (Medvedev et al., 2005). Besides that, acute high indole injection activated the vagus nerve, as was documented by eye blinking and dorsal vagal complex activation (Jaglin et al., 2018). On the other hand, chronic exposure to moderate doses of indole, achieved by monocolonization of GF rats with indole-producing *E. coli*, induced anxiety and depressive-like behavior, without affecting locomotor activity (Jaglin et al., 2018). Under these experimental settings, oxindole and isatin were not detected in the brain, but increased eye blinking frequency was evident, suggesting vagus activation. Therefore, this study showed that chronic indole overproduction is causally related to increased depressive and anxiety behaviors, which was partly mediated by vagus activation (Figure 2C).

Further, chronic moderate indole overproduction increased the detrimental effects of chronic stress on mice anxio-depressive phenotype (Mir et al., 2020). A mechanism *via* which indole achieved these effects included up-regulation of the adrenal catecholamine biosynthesis, one of the essential body adaptations to stressful events. On the other hand, the authors did not find any evidence of alterations in cytokine production or tryptophan metabolism along the serotonin and kynurenine pathways in mice exposed to indole.

One of the indole derivatives that could induce depressive behavior is IS, the major liver indole catabolite, as mentioned previously. After its production in the liver, it is absorbed by the circulation and excreted by the urine. However, because of its higher production, due to the increase of its precursor indole (King et al., 1966), or insufficient elimination by kidneys (Hobby et al., 2019), IS blood levels could rise. A study of chronic administration of high doses of IS to rats revealed that this indole metabolite accumulates in the brain, particularly in the brainstem, consequently increasing anhedonic behavior, emotional reactivity, and decreasing locomotion (Karbowska et al., 2020) (Figure 2C). These effects were accompanied by an altered concentration of brain monoamines, especially in the brainstem, and increased lipid peroxidation in CNS as a potential indicator of neuroinflammation (Karbowska et al., 2020). On the other hand, smaller doses of IS (i.e., 20 times smaller) exerted beneficial effects in the mice model of autoimmune encephalomyelitis by reducing markers of neuroinflammation (Rothhammer et al., 2016).

In the same study by Rothhammer et al. (2016), it was shown that several other indole derivatives, such as IPA and IAld, can also be neuroprotective in mice with autoimmune encephalomyelitis, and these effects were mediated *via* aryl hydrocarbon receptor (AhR) in astrocytes (Rothhammer et al., 2016). Interestingly, the probiotic cocktail comprising of 8 bacterial species (*B. bifidum W23, B. lactis W52, L. acidophilus W37, L. brevis W63, L. casei W56, L. salivarius W24, Lc. Lactis W19, Lc. Lactis W58*) elicited improvement of despair behavior in both control and high-fat diet fed rats, along with the increasing levels of circulating IPA (Abildgaard et al., 2017a). However, when the authors explored the direct effects of chronic IPA administration on rats, they did not observe any changes in anxiety and depression-like behavior (Abildgaard et al., 2017b). Although this study did not show antidepressive properties of IPA, it can still be considered as a potential candidate for the treatment of depression, for example, in other subtypes related to neuroinflammation. Alternatively, the combination of different indole metabolites could achieve desirable effects. Indeed, some *Bifidobacterium* and *Lactobacillus* spp. produce indole-related compounds, such as ILA and IAld, which might be implicated in their antidepressive properties, which could be a direction for future research (Roager and Licht, 2018) (Figure 2C).

One plausible mechanism that drives indole-mediated alterations of behavior is the activation of AhR, since indole and many of its products, either host- or microbiota-derived are known activators of this receptor (Hubbard et al., 2015; Agus et al., 2018). It is a ligand-activated transcription factor that plays a significant role in the microbiota-gut-brain axis by modulating the functioning of the immune system, the intestine, and the brain, as well as behavior (de la Parra et al., 2018; Gutiérrez-Vázquez and Quintana, 2018; Aguiniga et al., 2019; Obata et al., 2020). The role of AhR as a mediator of cross-talk between intestinal bacteria and the host in regulation of depression and anxiety behavior was shown in the study of Vicentini et al. (2021). They demonstrated that AhR knockout mice exhibited decreased helplessness behavior, similarly to mice depleted of their microbiota by antibiotic treatment. The despair behavior was normalized either by treatment with an AhR agonist or microbiota recolonization of antibiotic-treated mice (Vicentini et al., 2021), indicating that activation of AhR by microbial indolic metabolites is involved in the regulation of depressive behavior. However, whether peripheral and/or central activation of AhR takes part in achieving the observed effects remains unanswered. The other important question that is worth to be further explored is how the same receptor could be involved in both beneficial effects, for example, IPA, IAld, and lower doses of IS (Rothhammer et al., 2016), as well as aversive effects, for example, high concentrations of IS (Bobot et al., 2020; Karbowska et al., 2020), that are relevant for brain functioning and behavior.

Apart from AhR activation, other mechanisms could be implicated in behavioral effects of indoles. For example, isatin was shown to interact with several neurotransmitter systems, including inhibition of monoamine oxidase and activation of the 5-HT3 receptor (Bhattacharya et al., 1991; Abel, 1995; Medvedev et al., 2005). One other candidate for mediating the effects of indole and its derivatives might be the pregnane X receptor, a nuclear receptor present in many tissues, including the brain (Boussadia et al., 2018; Venu et al., 2019; Illés et al., 2020) and shown to be involved in processes that could be related to depressive behavior such as metabolism of neurosteroids and inflammation (Frye et al., 2013; Venkatesh et al., 2014). Further research is needed to reveal the contribution of these potential mechanisms in mediating indoles’ role in the pathophysiology of depression.

## Conclusion

In this review, we gave an overview of the complexity of bidirectional gut microbiota-brain communication with regard to tryptophan metabolism, whose disturbance is an essential factor contributing to the pathology of depression. Gut microbiota plays an important role in host tryptophan metabolism in various direct and indirect ways. Direct effects are achieved by bacterial metabolites such as short chain fatty acids (e.g., butyrate), hydrogen peroxide, indoles and its derivatives (Marin et al., 2017; Jaglin et al., 2018; Tian et al., 2019). Indirect effects of bacteria on tryptophan metabolism include modulation of the immune system, since pro-inflammatory cytokines are the potent inducers of IDO activity, driving the tryptophan metabolism down the kynurenine pathway. Therefore, probiotics with anti-inflammatory effects might have promising results in the treatment of depression (Liang et al., 2015; Sun et al., 2019). One bacterial species could exert its effects by several mechanisms (Marin et al., 2017; Xie et al., 2020). On the other hand, probiotics with combinations of several different bacteria could achieve desired effects by stimulating multiple mechanisms acting synergistically to alleviate depression (Kazemi et al., 2019). However, the potential of multibactrerial probiotic treatments, as well as prebiotics and symbiotics, as a way of restoring tryptophan metabolism and diminishing depressive behavior along the way, needs further exploration.

Preclinical studies gave useful information regarding the mechanisms of microbe–host interactions and promising results regarding the therapeutic effects of probiotics. Probiotics could provide an alternative or adjuvant therapy in treatment-resistant patients, balancing their tryptophan metabolism. However, more clinical data are needed to confirm such effects in humans. There are still numerous unknowns which limit the present potential of probiotic use in the treatment of depression. The complexity of individual microbiome communities, the genetic vulnerability of the host to depression, person’s lifestyle (such as diet and exposure to stress), and interactions of these factors could all impact the effects of probiotics in each individual. For example, as mentioned above, *L. reuteri* was shown to have anti-inflammatory and antidepressant effects in several studies, but it is also reported to cause depressive-like behavior *via* stimulation of inflammation in antibiotic pretreated mice (Wang et al., 2020). In addition, none of the studies examined long-term effects of probiotic treatments on tryptophan metabolism and depressive behavior, and whether these effects are dependent of transient or persistent colonization of the host gut. Also, although probiotics are regarded as generally safe, harmful effects may be under-reported in clinical trials and should be assessed more carefully in future studies (Lerner et al., 2019). In that context, adequate dosing of probiotics, as well as of their metabolites should be considered, since different concentrations showed to have different effects targeting heterogeneous mechanisms (Medvedev et al., 2005; Sun et al., 2019). Bearing in mind all these unknowns, carefully design future studies, with taking advantage of various “omic” techniques, may lead to simultaneous identification of multiple factors related to disturbed homeostasis of tryptophan metabolites in clinical depression, and choosing of adequate treatments for depressed patients.

## Author contributions

IL took part in designing the manuscript and figures, reviewing the literature, and writing the manuscript. SI and MM took part in reviewing the literature, and writing and revising the manuscript. MA took part in revising the manuscript. All authors contributed to the article and approved the submitted version.

## Abbreviations

3-HK: 3-hydroxykynurenine
5-HT: 5-hydroxytryptamine
5-HTP: 5-hydroxytryptophan
AANAT: arylalkylamine-*N*-acetyltransferase
AhR: aryl hydrocarbon receptor
ASMT: acetylserotonin *O*-methyltransferase
BBB: blood-brain barrier
CNS: central nervous system
DSS: dextran sulfate sodium
FMT: fecal microbiota transplantation
GF: germ free
HDAC: histone deacetylase
IAA: indole-3-acetic acid
IAld: indole-3-acetaldehyde
IDO: indoleamine 2,3-dioxygenase
IL10: interleukin 10
IL6: interleukin 6
ILA: indole-3-lactic acid
INFγ: interferon gamma
IPA: indole-3-propionic acid
IS: indoxyl-3-sulfate
KAT: kynurenine aminotransferase
KMO: kynurenine-3-monooxygenase
KYNA: kynurenic acid
LAT: L-amino acid transporter
miRNA: microRNA
NAD+: nicotinamide adenine dinucleotide
NMDA: *N*-methyl -D-aspartate
QUIN: quinolinic acid
SCN: suprachiasmatic nucleus
SERT: serotonin transporter
SNRI: serotonin norepinephrine reuptake inhibitor
SSRI: serotonin reuptake inhibitor
TDO: tryptophan 2,3-dioxygenase
TPH: tryptophan hydroxylase.
